# Physiologically-based pharmacokinetic modeling for single and multiple dosing regimens of ceftriaxone in healthy and chronic kidney disease populations: a tool for model-informed precision dosing

**DOI:** 10.3389/fphar.2023.1200828

**Published:** 2023-07-20

**Authors:** Fawaz Alasmari, Mohammed S. Alasmari, Hussa Mubarak Muwainea, Hatun A. Alomar, Abdullah F. Alasmari, Sary Alsanea, Aws Alshamsan, Muhammad F. Rasool, Faleh Alqahtani

**Affiliations:** ^1^ Department of Pharmacology and Toxicology, College of Pharmacy, King Saud University, Riyadh, Saudi Arabia; ^2^ Department of Pharmaceutics, College of Pharmacy, King Saud University, Riyadh, Saudi Arabia; ^3^ Department of Pharmacy Practice, Faculty of Pharmacy, Bahauddin Zakariya University, Multan, Pakistan

**Keywords:** PBPK, Pk-sim^®^, CKD, ceftriaxone, PK parameters

## Abstract

**Introduction:** Ceftriaxone is one of commonly prescribed beta-lactam antibiotics with several label and off-label clinical indications. A high fraction of administered dose of ceftriaxone is excreted renally in an unchanged form, and it may accumulate significantly in patients with impaired renal functions, which may lead to toxicity.

**Methods:** In this study, we employed a physiologically-based pharmacokinetic (PBPK) modeling, as a tool for precision dosing, to predict the biological exposure of ceftriaxone in a virtually-constructed healthy and chronic kidney disease patient populations, with subsequent dosing optimizations. We started developing the model by integrating the physicochemical properties of the drug with biological system information in a PBPK software platform. A PBPK model in an adult healthy population was developed and evaluated visually and numerically with respect to experimental pharmacokinetic data. The model performance was evaluated based on the fold error criteria of the predicted and reported values for different pharmacokinetic parameters. Then, the model was applied to predict drug exposure in CKD patient populations with various degrees of severity.

**Results:** The developed PBPK model was able to precisely describe the pharmacokinetic behavior of ceftriaxone in adult healthy population and in mild, moderate, and severe CKD patient populations. Decreasing the dose by approximately 25% in mild and 50% in moderate to severe renal disease provided a comparable exposure to the healthy population. Based on the simulation of multiple dosing regimens in severe CKD population, it has been found that accumulation of 2 g every 24 h is lower than the accumulation of 1 g every 12 h dosing regimen.

**Discussion:** In this study, the observed concentration time profiles and pharmacokinetic parameters for ceftriaxone were successfully reproduced by the developed PBPK model and it has been shown that PBPK modeling can be used as a tool for precision dosing to suggest treatment regimens in population with renal impairment.

## 1 Introduction

Ceftriaxone is a third-generation cephalosporin antibiotic with a broad-spectrum activity against a wide range of microbial infections. Mechanistically, ceftriaxone acts as a bactericidal agent by inactivating penicillin-binding proteins in the outer cytoplasmic membrane and inhibiting bacterial cell wall synthesis (Fontana et al., 1998; Kocaoglu and Carlson, 2015). Due to its physicochemical characteristics, ceftriaxone can cross different biological barriers and penetrate deep into other systemic tissues, including the blood-brain barrier. Thus, it has been approved to treat infections that affect various body organs, including the central nervous system, lung tissue, skin and soft tissue, bone and joints, and urinary tract infections. (Steele, 1984; Le Turnier et al., 2019). It is well tolerated and exhibits a good safety profile at the standard doses with a predictable pharmacokinetic behavior. Clinically, ceftriaxone can be used as empirical therapy before the culture susceptibility is available, and then treatment protocol can be converted to a pathogen-specific therapy. According to the literature, 33%–67% of ceftriaxone is eliminated in unchanged form through the kidney, while the remaining fraction is excreted through the biliary system (Patel and Kaplan, 1984). The protein bound fraction of ceftriaxone in the plasma was estimated to be 60%–95% (Popick et al., 1987). Given that ceftriaxone exhibits very low bioavailability after oral administration (<1%), it is only administered parenterally as intravenous or intramuscular injections (Nau et al., 2010).

One of the most determinants of drug kinetic behavior is the kidney’s functional status, and pharmacokinetic parameters are highly affected in patients with chronic kidney disease (CKD) (Rowland Yeo et al., 2011; Velenosi and Urquhart, 2014). Therefore, patients with CKD should be closely monitored, especially for renally excreted drugs (Tan et al., 2018). Appropriate dose selection according to the functional status of the kidneys is necessary to avoid drug build-up in the body, which may increase the risk of toxicity (Trotman et al., 2005; Patel et al., 2010; Morales-Alvarez, 2020). According to recently published data (Lacroix et al., 2021), severe adverse reactions, including deaths, convulsions, hallucinations, and other brain toxicities, were seen in patients treated with ceftriaxone. It has been mentioned that these toxic events were attributed to ceftriaxone. A recommendation has been given to clinicians to avoid this danger, especially in patients with renal impairment. Notably, the plasma level of ceftriaxone was found to be above the toxic limits in many patients. Therefore, proper administration of ceftriaxone is necessary to achieve the optimal benefit and prevent potential toxicities by maintaining ceftriaxone plasm levels within therapeutic ranges and avoiding any accumulation (Aloy et al., 2020; Chahine, 2022).

Physiologically-based pharmacokinetic (PBPK) models are mathematical and quantitative in nature, and they are developed to predict drug absorption, distribution, metabolism, and excretion (ADME). Modeling and simulation are now standard practices in the drug development process with the ultimate goal of improving the efficacy and safety of drugs (Li et al., 2017; Taskar et al., 2020; Verscheijden et al., 2020; Wang et al., 2021). In early clinical trials, several patient populations are inaccessible, either ethically or for other reasons, and they cannot be included in clinical studies, such as pediatrics, pregnant women, or patients with chronic renal and hepatic diseases. PBPK models are one of the alternative approaches that are authenticated to predict drug exposure in those populations with subsequent dosing suggestions based on individualized physiological needs. PBPK models have been used in various fields, such as human health risk assessment, environmental risk assessment, and drug discovery and development. They are considered a powerful approach for detecting the concentration of xenobiotics in tissues of interest, and they facilitate the *in vitro* to *in vivo* extrapolation. Accurate parametrization of the models is very important for the extrapolation and application of the PBPK model (Thiel et al., 2015).

According to clinical indications, ceftriaxone has been recommended to be given in doses of 250 mg, 500 mg, 1 g, or 2 g daily or two times a day for a specific period of time. A previous study demonstrated alterations in the PK parameters of ceftriaxone in patients with mild, moderate, and severe renal impairment (Patel et al., 1984). Except for patients on dialysis, the study did not recommend dosing modification as long as no more than 2 g/day was prescribed. However, the recently published data about ceftriaxone-induced toxicity mentioned that the median dose that intoxicated patients administered was 1.7 g/day (Lacroix et al., 2021). Therefore, using the PBPK method is highly suitable in the current situation to provide a quick overview of drug exposure in the CKD population and validate the PK information of ceftriaxone that might be required to avoid the potential of adverse events. Thus, the current study was performed to evaluate the need for ceftriaxone dosage adjustment in patients at different stages of renal insufficiency.

## 2 Materials and methods

### 2.1 PBPK software

PK-Sim software (version 9; Open System Pharmacology [OSP] Suite (https://www.open-systems-pharmacology.org) was used to simulate ceftriaxone concentrations over time in the plasma. The simulation was created based on the interplay between physicochemical, physiological, and biochemical factors (Cole et al., 2020). The PK-Sim platform was designed to be consisting of several building blocks while taking into account several external and internal factors that may influence the PK of the drugs (Farhan et al., 2022). Structurally, the model is represented by several biological compartments correlated to each other utilizing arterial and venous blood circulations. The observed data of ceftriaxone plasma concentration *versus* time was digitized using Get-Data Graph Digitizer^®^ (version 2.26), according to a previous study (Wojtyniak et al., 2020).

### 2.2 Literature search

A literature search was conducted through different electronic databases, including MEDLINE, EMBASE, and Google Scholar, to retrieve clinical PK studies to be used for the PBPK model development process. We included studies that evaluated intravenous administration of ceftriaxone in adult healthy and CKD patient populations. In order to use clinical PK data in the development and verification processes of PBPK models, concentration *versus* time profiles have to be available and described with rich-sampling scheme and uniform sampling times, which typically conducted in early phase of drug development. Clinical PK studies used for developing and evaluating the ceftriaxone PBPK model are shown in Table 1. The physicochemical properties of ceftriaxone (Table 2) that were used for developing this model were obtained from PubChem (Kim et al., 2021), DrugBank (Wishart et al., 2018a), and the Human Metabolome Database (Wishart et al., 2018b).

**TABLE 1:** Clinical studies that were used for development and evaluation of the ceftriaxone PBPK model.

Dose	Inf. Time	N	Female (n)	Age (year)	Weight (kg)	Reference
Single dosing regimens of ceftriaxone in adult healthy population						
500 mg	20 min	10	5	29.5 (22–43)	65.2 (49–75)	Borner et al. (1985)
2000 mg	20 min	10	5	29.5 (22–43)	65.2 (49–75)	Borner et al. (1985)
500 mg	30 min	12	2	36 (21–47)	74.1 (53–94.8)	Patel et al. (1981)
1,000 mg	30 min	12	2	36 (21–47)	74.1 (53–94.8)	Patel et al. (1981)
2000 mg	30 min	12	2	36 (21–47)	74.1 (53–94.8)	Patel et al. (1981)
1,000 mg	30 min	30	5	34.5 (18–65)	79.3 ± 11.3	Harb et al. (2010)
500 mg	3 min	6	0	(21–37)	78.2	Seddon et al. (1980),^a^
500 mg	Bolus	6	N/A	N/A	N/A	Stoeckel (1981),^a^
1,500 mg	Bolus	6	N/A	N/A	N/A	Stoeckel (1981),^a^
3,000 mg	Bolus	6	0	(23–29)	N/A	McNamara et al. (1982),^a^
Multiple dosing regimens of ceftriaxone in adult healthy population						
500 mg q12 h	30 min	12	N/A	29 (19–45)	70 (57–99)	Pollock et al. (1982)
1,000 mg q12 h	30 min	12	N/A	31 (21–51)	73 (56–99)	Pollock et al. (1982)
2000 mg q12 h	30 min	12	N/A	33 (20–51)	74 (54.5–113.5)	Pollock et al. (1982)
2000 mg q24 h	30 min	8	N/A	28.3 (21–46)	71.8 (62.5–75.2)	Pollock et al. (1982)
Single dosing regimen of ceftriaxone in CKD patient population with various degree of severity						
1,000 mg	15 min	30	N/A	52.6 (21–75)	65.9 (51.8–103)	Patel et al. (1984)

^a^ Data from these studies were used as external test dataset.

**TABLE 2:** Parameters that were used for developing the ceftriaxone PBPK model.

	Unit	Input value	Reported value	Reference of reported value
Physicochemical properties				
Molecular weight	g/mol	554.58	554.58	Wishart et al. (2018b)
Lipophilicity	Log	– 1.7	– 1.7	Wishart et al. (2018b)
pKa (acid)		2.7	2.7	Wishart et al. (2018a)
Distribution				
Partition coefficient model	Poulin/Theil model			PK-Sim
Cellular permeability model	PK-Sim standard model			
Fraction unbound	%	12.5^a^	5–40	Popick et al. (1987)
Elimination				
Biliary clearance	mL/min	3.67^a^	1–13	Arvidsson et al. (1982)
Renal clearance	L/hour	0.657^a^	0.32–0.73	Kim et al. (2021)

^a^ Value has been identified using parameter identification feature supplied with the PK-Sim software.

### 2.3 Designing PBPK models for adult healthy and CKD patient populations

**FIGURE 1:**
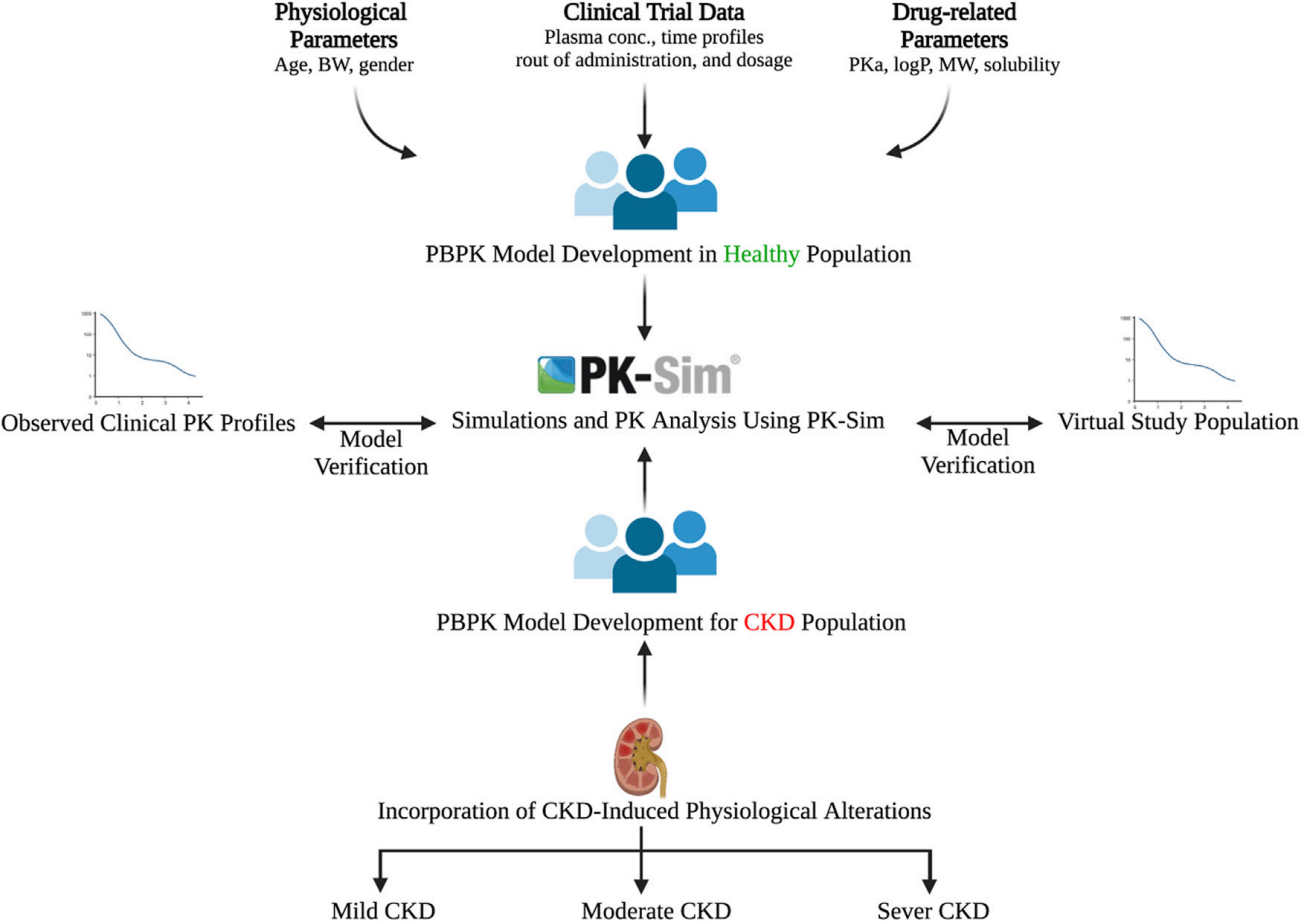
Workflow for developing the ceftriaxone PBPK model. The figure was created with BioRender.com with agreement number HZ250T3MDL.

The general guideline of the European Medicines Agency was followed for PBPK model development and evaluation (Luzon et al., 2017). Previous preliminary PBPK models for several renally cleared drugs, including ceftriaxone in Chinese pregnant women, were identified and reviewed to have better initial estimates for the model parameters (Song et al., 2020). A general schematic workflow for the overall process of developing the ceftriaxone PBPK model is depicted in Figure 1, and model parameters are defined in Table 2. We started the modeling by integrating drug-specific parameters (e.g., MW. logP, pKa), and trial design-specific parameters (e.g., administration protocol, route of administration, dose, and dosing frequency) with the predefined biological system-specific parameters in the PK-Sim. The fraction of drug unbound in plasma was used in conjunction with the physicochemical characteristics to quantify tissue partitioning, membrane permeation, and other biological passive processes. Given that ceftriaxone is administered parenterally, the model was established after intravenous administration, and thus, only information on distribution and elimination properties were gathered. The distribution phase was modeled by taking into account partition coefficients and cellular permeability. The partition coefficient was calculated automatically using Poulin/Theil method, whereas cellular permeability was calculated using the PK-Sim standard method. Renal clearance was modeled by choosing renal plasma clearance as a process type in the PK-Sim software. When choosing this process type, the PK-Sim software automatically calculates the renal clearance by taking into account the experimental value for kidney plasma clearance and fraction unbound of the drug (fu) in plasma. Biliary clearance was added to the model based on the experimental values as illustrated in Table 2. Once the model was developed and evaluated based on adult healthy population demographics and data, the model was used to predict the exposure in CKD patients by taking into account the accompanying pathophysiological alterations of CKD with various degrees of severity based on the previous findings, as identified in Table 3. The physiological parameters that have been modified in the CKD are creatinine clearance, kidney volume, renal perfusion, and hematocrit (Malik et al., 2020). These parameters directly impact renal clearance. The effect of the CKD on the fraction unbound was accounted for by using a plasma protein scaling factor that is provided with the PK-Sim software. This scalar is recommended to be used if the changes in plasma protein concentrations and, as a result, plasma protein binding are expected due to pathological conditions such as renal failure. For biliary clearance, no parameter was accounted for in the CKD modeling. Thus, it is assumed that CKD patients have intact biliary system.

**TABLE 3:** Pathophysiological changes associated with the severity of the CKD according to Malik et al., 2020 (Malik et al., 2020).

Parameter	Stage of CKD		
	Mild 30–60 mL/min/1.73 m^2^	Moderate 15–30 mL/min/1.73 m^2^	Severe <15 mL/min/1.73 m^2^
Creatinine clearance	41–74	18–30	5–14
Kidney volume	132 mL	92 mL	76 mL
Renal perfusion	176 mL/min/100 g	97 mL/min/100 g	75.35 mL/min/100 g
Hematocrit	0.44	0.40	0.34
Correction factor^a^	1.45	2.20	2.50

^a^ Correction factor for the effect of the CKD, on the content of plasma proteins as described by Malik and colleagues (Malik et al., 2020).

### 2.4 Evaluation of the PBPK model and predictability assessment

The PBPK model was evaluated according to previously published guidelines (Kuepfer et al., 2016). The model’s performance was evaluated visually and considered successful when the simulated and experimental PK findings fell within the 5th and 95th percentiles. A numerical evaluation according to the mean fold error (MFE) and mean square root of error (RMSE) was used as indicators to examine how much the predicted values deviated from the observed values (Eqs 2, 3). As described in many previous constructed models, the acceptable error range for the predicted to observed values was determined to be within a two-fold range.

$$Ratio=\frac{Predicted\;value\;of\;PK\;parameter}{Observed\;value\;of\;PK\;parameter}$$ (1)

$$MFE=\frac{Mean\;of\;Predicted\;Values}{Mean\;of\;Observed\;Values}$$ (2)

$$RMSE=\sqrt{\frac{\sum_{1}^{N}{\left(Observed\;value-Predicted\;value\right)}^{2}}{N}}$$ (3)

## 3 Results

### 3.1 Development of a PBPK model in adult healthy population after single and multiple dosing regimens

**FIGURE 2:**
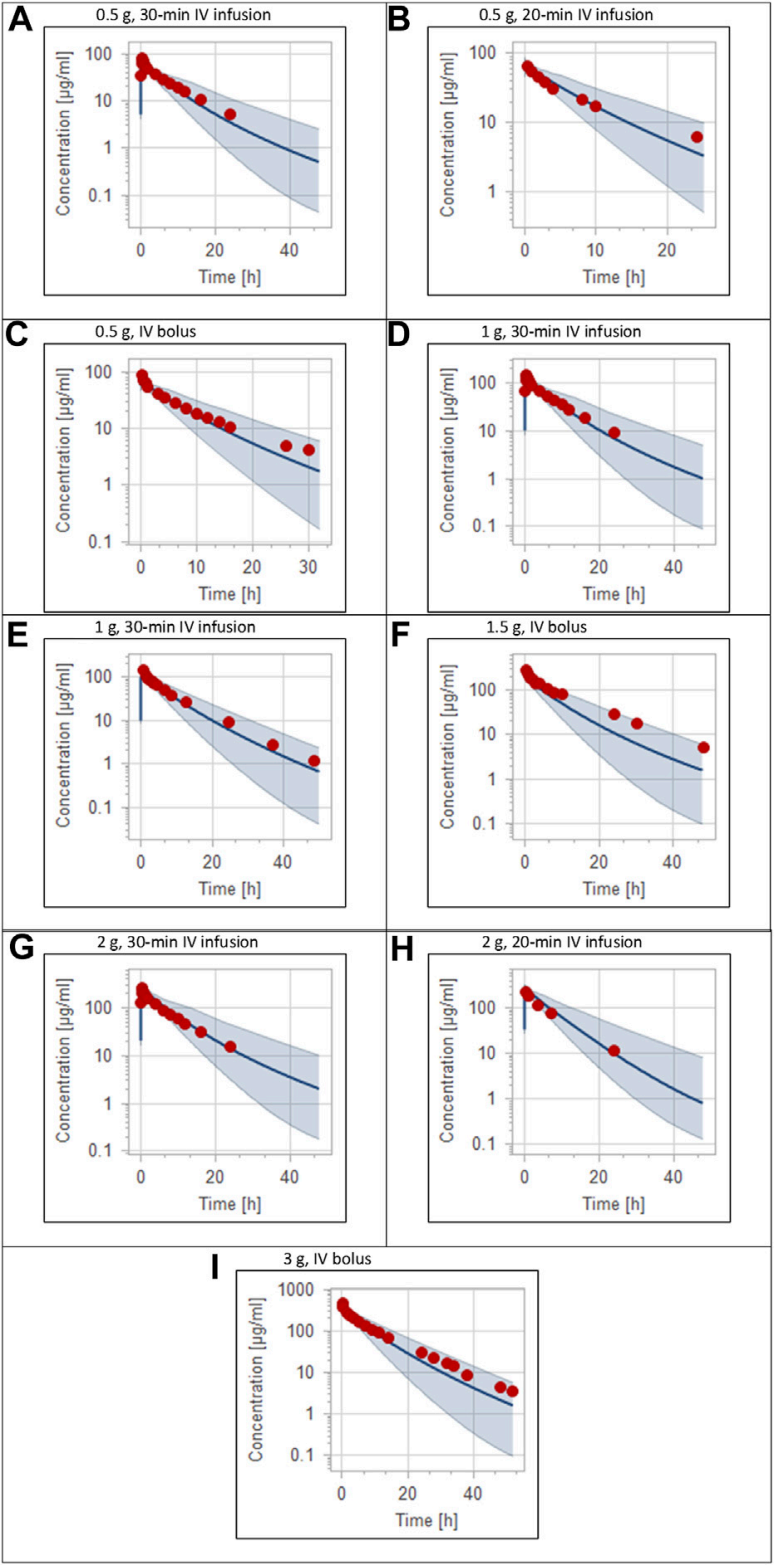
Plasma concentration-time profiles for intravenous administration of ceftriaxone in adult healthy population. **(A)** 0.5 g (Patel et al., 1981), **(B)** 0.5 g (Borner et al., 1985), **(C)** 0.5 g (Seddon et al., 1980), **(D)** 1 g (Patel et al., 1981), **(E)** 1 g (Harb et al., 2010), **(F)** 1.5 g (Stoeckel, 1981), **(G)** 2 g (Patel et al., 1981), **(H)** 2 g (Borner et al., 1985), **(I)** 3 g (McNamara et al., 1982). The 5th to 95th percentile prediction range is shown as a shaded area, and observed data points are indicated as red circles; simulated means are indicated as solid lines.

**FIGURE 3:**
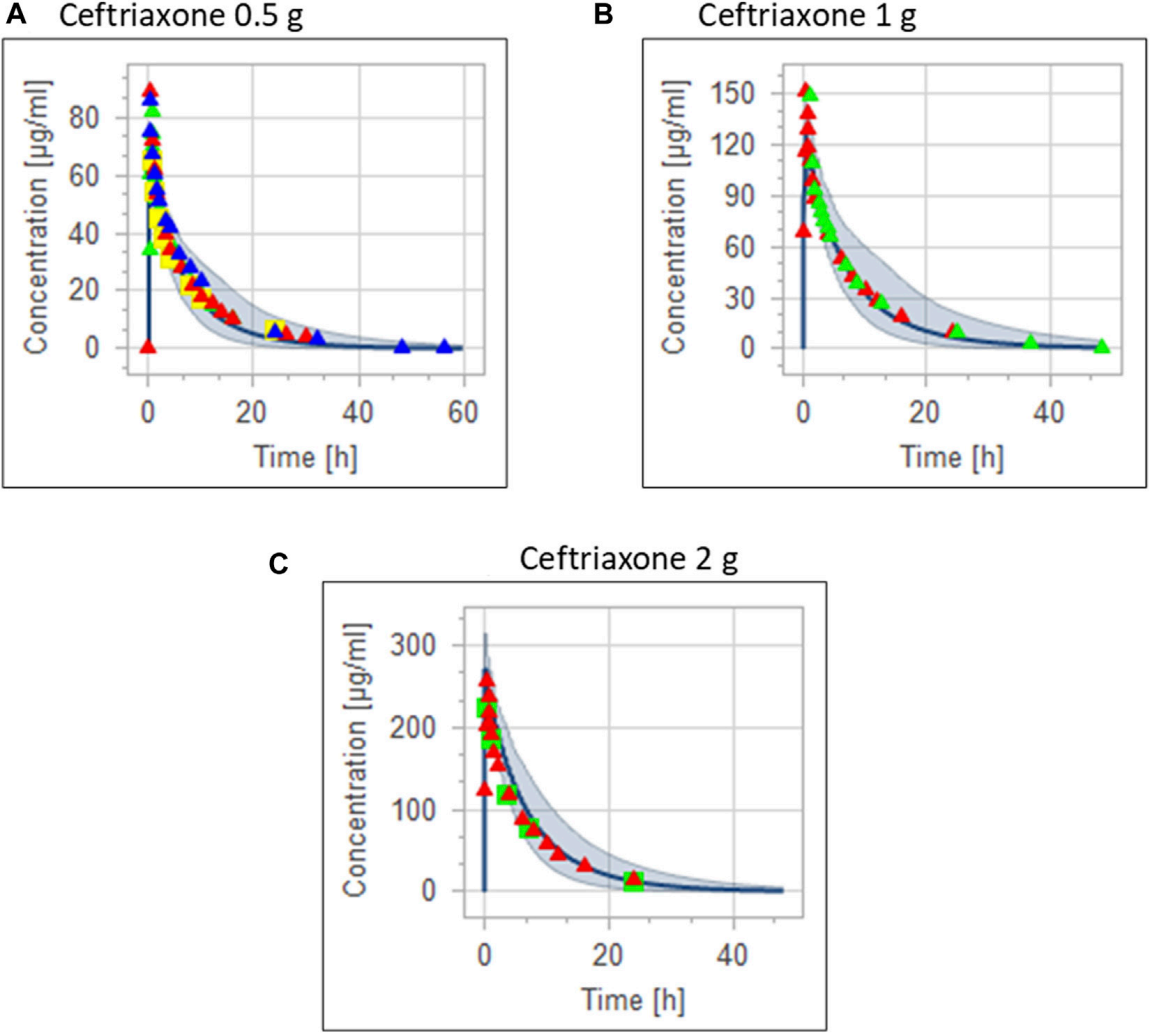
Combining the same doses of ceftriaxone from different studies for visual verification of the PBPK model in adult healthy population. Observed data are depicted as colored shapes, while solid line and shaded areas representing the prediction mean and 5th to 95th prediction range, respectively. **(A)** after administering 0.5 g ceftriaxone. **(B)** after administering 1 g ceftriaxone. **(C)** after administering 2 g ceftriaxone.

**FIGURE 4:**
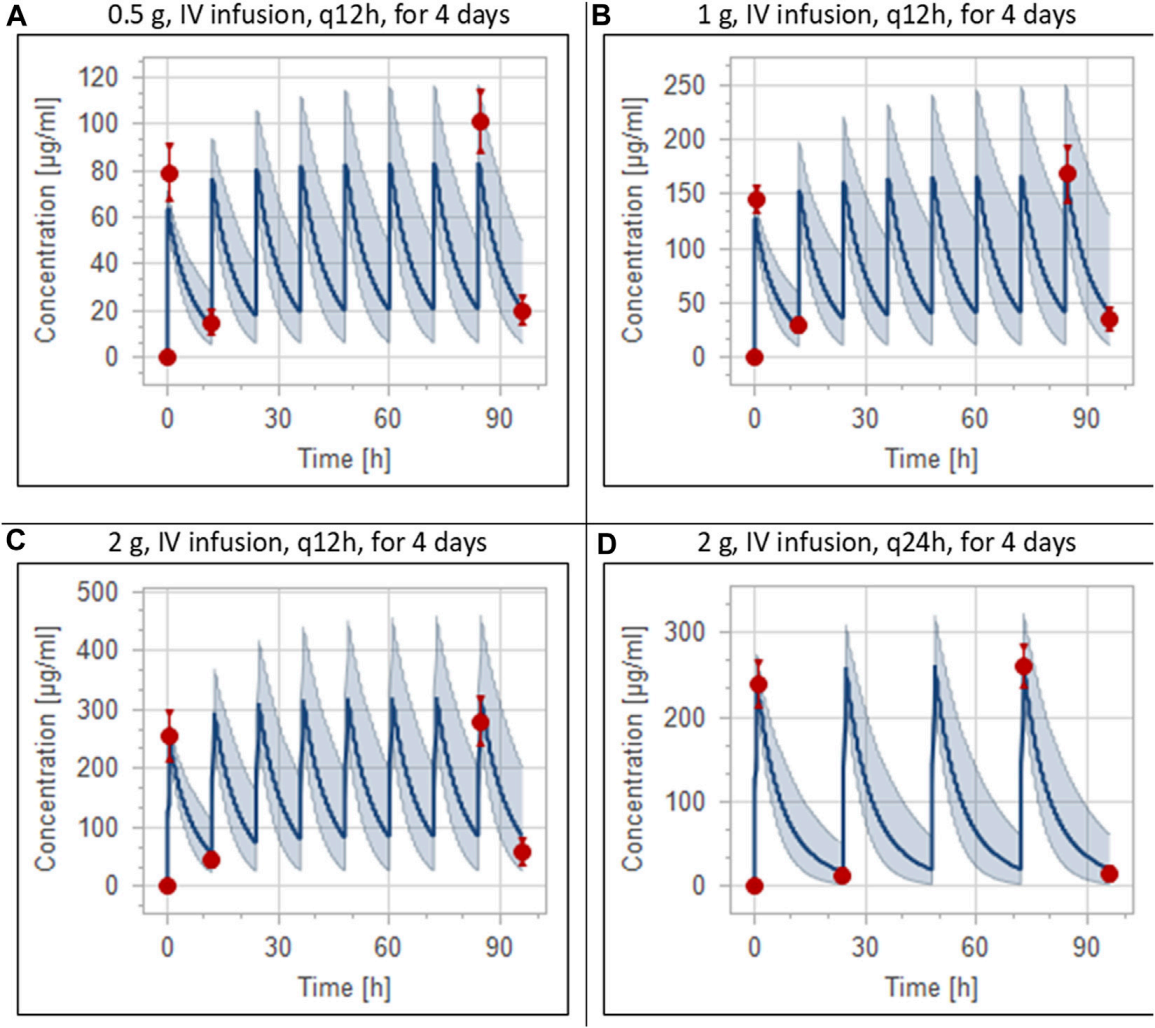
Simulation of concentration *versus* time profiles of multiple dosing regimens of ceftriaxone in adult healthy populations. Observed peak and trough concentrations (Pollock et al., 1982) are depicted as colored circles, while solid lines and shaded areas representing the prediction mean and 5th to 95th prediction interval, respectively. **(A)** after administering 0.5 g ceftriaxone intravenous infusion every 12 h for 4 days. **(B)** after administering 1 g ceftriaxone intravenous infusion every 12 h for 4 days. **(C)** after administering 2 g ceftriaxone intravenous infusion every 12 h for 4 days. **(D)** after administering 2 g ceftriaxone intravenous infusion every 24 h for 4 days.

The clinical PK studies used for the model development and evaluation are summarized in Table 1. Data from three studies were used as external test dataset (Seddon et al., 1980; Stoeckel, 1981; McNamara et al., 1982) and they were only used for the visual verification of the model. Using a virtual human population of 100 healthy individuals, we developed a population PBPK model after intravenous administration for a wide range of single and multiple dosing regimens of ceftriaxone with subsequent model validation processes with respect to the observed data from the clinical PK studies. The model’s performance was evaluated visually as demonstrated in Figures 2–4, where most of the observed time points were included within the 5th to 95th prediction interval. For single dosing regimens, the developed model was further evaluated by comparing the ratio of predicted-to-observed values for the PK parameters, including AUC, Cmax, T½, and clearance (CL). All the calculated predicted-to-observed PK data were within the predefined acceptable two-fold range as demonstrated in Table 4. The MFE of the AUC, Cmax, T½, and CL were 1.01, 0.90, 1.20, and 0.98, respectively. Because that peak and trough concentrations are very important PK parameters in the dosing adjustment for antibiotics, the PBPK model for multiple dosing regimens was evaluated based on the ability of the model to reproduce the maximum and minimum concentrations after the first dose and at steady state. Four days were required for ceftriaxone to reach steady state concentration which is in the line with the reported values (Pollock et al., 1982). We evaluated the ability of the model to capture the peak (Cmax) and trough concentrations (Cmin) at the first day and at the steady state visually (Figure 4) and numerically by comparing the ratio of predicted-to-observed values for the corresponding parameters. All the calculated predicted-to-observed values were within the predefined acceptable two-fold range as demonstrated in Table 5.

**TABLE 4:** Predicted-to-observed ratios of PK parameters of ceftriaxone in the adult healthy population.

PK parameter		Predicted	Observed	Ratio
0.5 g I.V. infusion (Patel et al., 1981)				
AUC [μg/mL*h]		490.1	551 (462–737)	0.89
Cmax [μg/mL]		63.5	82	0.77
T½ [h]		8.34	6.30 (5.45–7.75)	1.32
CL [mL/min]		17.04	15.48 (11.3–19.83)	1.10
0.5 g I.V. infusion (Borner et al., 1985)				
AUC [μg/mL*h]		490.1	551 (462–737)	0.89
Cmax [μg/mL]		63.5	82	0.77
T½ [h]		8.34	6.30 (5.45–7.75)	1.32
CL [mL/min]		17.04	15.48 (11.3–19.83)	1.10
0.5 g I.V. infusion (Borner et al., 1985)				
AUC [μg/mL*h]		517.3	549 ± 125	0.94
Cmax [μg/mL]		74.23	83.8 ± 40.1	0.89
T½ [h]		7.20	9.87 ± 2.22	0.73
CL [mL/min]		16.11	16 ± 4.3	1.01
1 g I.V. infusion (Patel et al., 1981)				
AUC [μg/mL*h]		988	1,006 (764–1,238)	0.98
Cmax [μg/mL]		127	150.7	0.84
T½ [h]		8.37	6.13 (5.0–7.24)	1.37
CL [mL/min]		17.03	16.78 (13.47–21.82)	1.01
1 g I.V. infusion (Harb et al., 2010)				
AUC [μg/mL*h]		933	1,085.8 ± 187.5	0.86
Cmax [μg/mL]		120.86	150 ± 19.9	0.81
T½ [h]		8.89	8.25 (6.03–10.4)	1.08
CL [mL/min]		17.87	15.86	1.13
2 g I.V. infusion (Patel et al., 1981)				
AUC [μg/mL*h]		1974.5	1,703 (1,308–2,055)	1.16
Cmax [μg/mL]		254	256.9	0.99
T½ [h]		8.86	5.82 (4.73–6.84)	1.68
CL [mL/min]		16.90	19.83 (16.22–25.48)	0.85
2 g I.V. infusion (Borner et al., 1985)				
AUC [μg/mL*h]		1,908.44	1,565 ± 328	1.22
C_max [μg/mL]		278	258 ± 38.4	1.08
T½ [h]		6.58	6.4 ± 1.07	1.03
CL [mL/min]		17.47	22.1 ± 5.0	0.79
Predictability assessment				
	AUC	C_max	T½	CL
MFE	1.01	0.90	1.20	0.98
RMSE	191.5	19.4	2.08	2.47

**TABLE 5:** Comparison of predicted and observed (Pollock et al., 1982) peak and trough concentrations of ceftriaxone after multiple dosing regimens.

	Peak concentration^a^			Trough concentration^a^		
	Simulated	Observed	FE	Simulated	Observed	FE
0.5 g intravenous ceftriaxone q12 h in healthy population						
Day 1	64 (57–71)	79 (64–102)	1.23	14 (6–28)	15 (8.6–24)	1.07
Day 4	84 (66–117)	101 (77–117)	1.20	21 (7–50)	20 (14–28)	1.05
0.5 g intravenous ceftriaxone q12h in severe CKD population						
Day 1	63 (43–127)			31 (20–57)		
Day 4	130 (80–240)			68 (36–146)		
1 g intravenous ceftriaxone q12h in healthy population						
Day 1	128 (113–143)	145 (130–160)	1.13	28 (12–56)	30 (23–42)	1.07
Day 4	167 (133–234)	168 (132–213)	1.01	42 (13–101)	35 (23–58)	1.2
1 g intravenous ceftriaxone q12h in severe CKD population						
Day 1	136 (85–259)			66 (40–113)		
Day 4	279 (160–480)			149 (73–295)		
2 g intravenous ceftriaxone q12h in healthy population						
Day 1	244 (216–273)	255 (184–338)	1.04	58 (24–114)	45 (29–64)	1.30
Day 4	322 (252–459)	280 (214–346)	1.15	87 (28–205)	59 (37–111)	1.5
2 g intravenous ceftriaxone q12h in severe CKD population						
Day 1	263 (166–494)			135 (81–230)		
Day 4	546 (315–945)			301 (146–590)		
2 g intravenous ceftriaxone q24h in healthy population						
Day 1	244 (212–270)	239 (198–278)	1.02	17 (3–50)	13 (7–23)	1.31
Day 4	263 (222–323)	260 (216–281)	1.01	21 (3–63)	15 (7–27)	1.40
2 g intravenous ceftriaxone q24h in severe CKD population						
Day 1	263 (167–487)			72 (36–130)		
Day 4	361 (215–631)			107 (46–225)		

^a^ The simulated and observed values (Pollock et al., 1982) represent the mean and the values between parentheses is the range. FE: fold error, CKD: chronic kidney disease.

### 3.2 Development of the PBPK model in different stages of CKD after ceftriaxone intravenous administration

**FIGURE 5:**
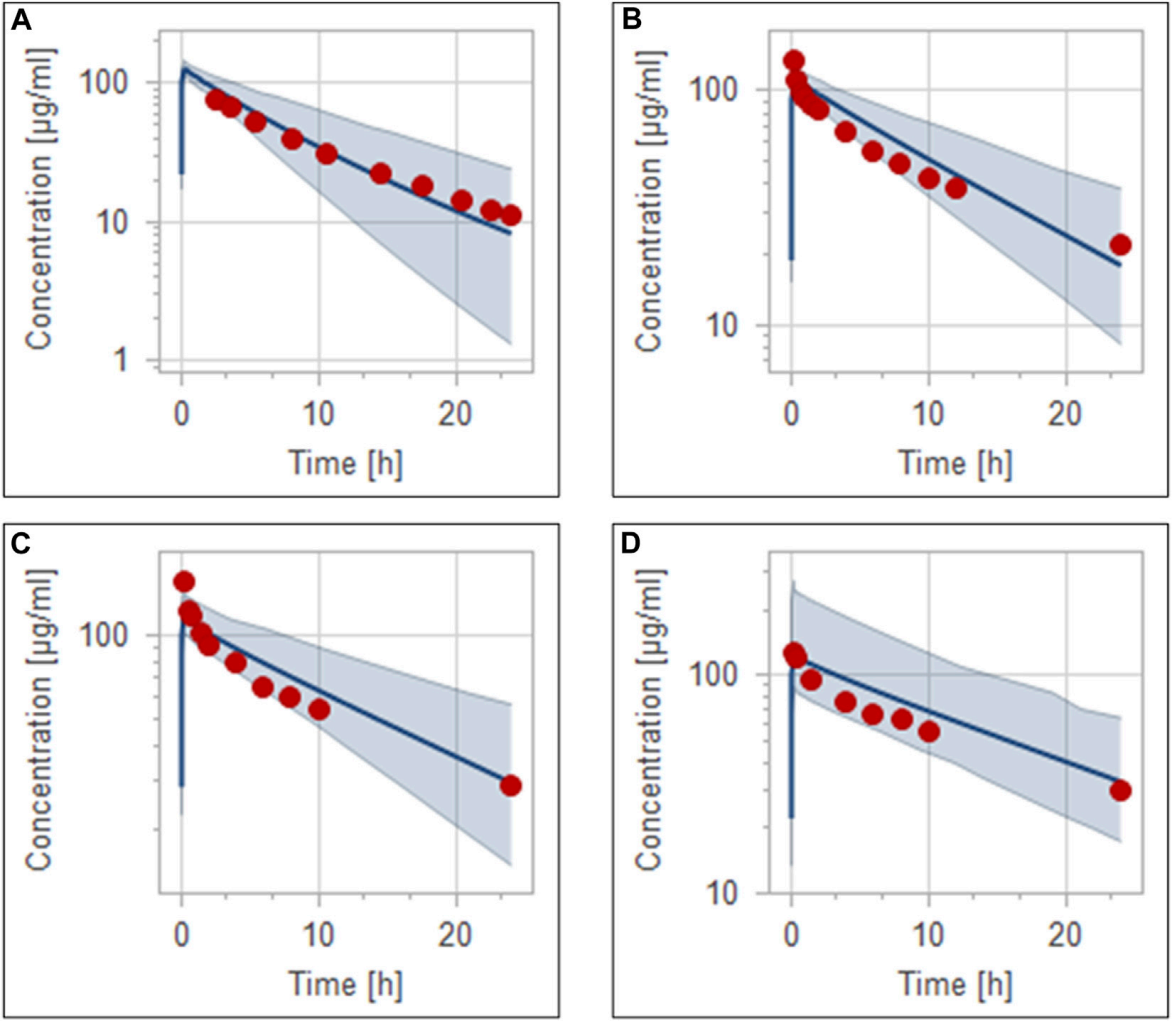
Simulation of concentration *versus* time profiles of 1 g intravenous infusion of ceftriaxone in healthy population **(A)**, mild renal impairment population **(B)**, moderate renal impairment population **(C)**, and severe renal impairment population **(D)**. Observed data are depicted as colored circles, while solid line and shaded areas representing the prediction mean and 5th to 95th prediction range, respectively.

Pathophysiological changes associated with the CKD severity stages were obtained from a previous study (Malik et al., 2020). They included creatinine clearance, kidney volume, renal perfusion, and hematocrit. The altered values were incorporated into the PK-Sim to predict drug concentration in the CKD patient population with varying degrees of severity. A previous clinical PK study investigated the effects of CKD on PK parameters of 1 g ceftriaxone after intravenous administration, and it was used for the CKD PBPK model evaluation (Patel et al., 1984). After generating a virtual CKD population with different degrees of severity based on the previously mentioned CKD-specific physiological alterations, the model was used to predict PK parameters of ceftriaxone in case of mild, moderate, and severe renal impairment. The model successfully captured the observed data, as represented graphically in Figure 5. Moreover, the model was verified according to the predicted-to-observed ratios of the PK parameters (AUC, T½, and CL). The fold errors of all PK parameters of ceftriaxone in the healthy model and the mild, moderate, and severe renal impairment disease models were within the predefined acceptable two-fold error range (Table 6). Based on the simulation of multiple dosing regimens in severe CKD population (supplementary, Figure 1S), it has been found that accumulation of 2 g every 24 h (factor of 1.50) is lower than the accumulation of 1 g every 12 h (factor of 2.30) dosing regimen (Table 7). This may be an indicator of the applicability of the high-dose extended-interval protocol in patient with deterioration in renal function, in comparison to low-dose multiple interval treatment protocol.

**TABLE 6:** PK analysis of time profiles for CKD patients.

PK parameter	Data type	Healthy	CKD		
			Mild	Moderate	Severe
AUC [μg/mL*h]	Predicted	1,037	1,454.63	2,011.93	2,211.58
	Observed	894.77	1,558.10	1,970.87	2,025.89
	Fold error	1.16	1.07	1.02	1.09
T½ [h]	Predicted	7.67	9.40	12.88	13.08
	Observed	8.96	14.69	15.30	15.42
	Fold error	1.17	1.56	1.19	1.18
Clearance [mL/min]	Predicted	16.10	11.45	8.30	7.54
	Observed	20.05 ± 3.15	11.75 ± 4.20	8.82 ± 1.62	10.05 ± 2.95
	Fold error	1.25	1.03	1.06	1.33

**TABLE 7:** Ratio of trough concentrations at steady state to the trough concentrations after the first application, as estimation of accumulation index.

Regimen	Healthy		CKD
	Simulated	Observed	Simulated
0.5 g, q12h, for 4 days	1.50	1.33	2.20
1 g, q12h, for 4 days	1.50	1.20	2.30
2 g, q12h, for 4 days	1.50	1.31	2.23
2 g, q24h, for 4 days	1.24	1.20	1.50

### 3.3 Dosing adjustment of ceftriaxone in subjects with different stages of CKD

**FIGURE 6:**
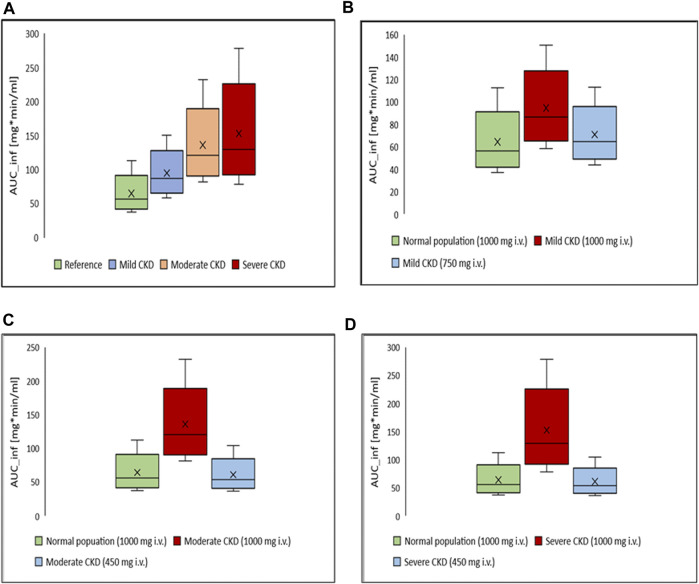
Box-whisker plots for the effect of various degrees of CKD on the exposure of ceftriaxone in comparison to healthy individuals, with subsequent dosing optimizations. **(A)** Comparison, in term of AUC, between healthy (reference) and CKD populations with various degrees of severity after administration of 1,000 mg intravenous ceftriaxone. **(B)** AUC of ceftriaxone after the dose was decreased to 750 mg in mild CKD, in comparison to healthy subjects administered 1,000 mg ceftriaxone. **(C)** AUC after the dose was decreased to 450 mg in moderate CKD, in comparison to normal subjects administered 1,000 mg ceftriaxone. **(D)** AUC after the dose was decreased to 450 mg in severe CKD, in comparison to normal subjects administered 1,000 mg ceftriaxone.

Box-whisker plots for the AUC of 1 g intravenous ceftriaxone in patients with various degrees of CKD compared to healthy individuals as a reference are shown in Figure 6. It has been shown that the exposure increased as CKD progressed. In gradual dosing reduction simulation trials, we found that 750 mg (25% reduction in the initial dose) and 450 mg (55% reduction in the initial dose) are comparable in exposure to the reference healthy simulated population for mild CKD population, and for moderate to severe CKD population, respectively (Figure 6 B, C, and D).

## 4 Discussion

Ceftriaxone is a highly effective antimicrobial agent used to treat various infections (Epstein et al., 1982; Cleeland and Squires, 1984). The PK of ceftriaxone has been extensively studied in human and animal models (Patel et al., 1981; Rebuelto et al., 2003; Buragohain et al., 2021); however, the literature is still incomplete in many key areas, including special populations such as CKD patients. Clinically, it has been demonstrated that ceftriaxone clearance is decreased severely in patients with creatinine clearance lower than 5 mL/min/1.73 m^2^ (Patel et al., 1984). Although ceftriaxone is prescribed with caution in patients with CKD, specific dosing recommendations based on renal function have not been provided (Munar et al., 2007). In this study, we aimed to explore the effects of renal failure on the pharmacokinetic parameters of ceftriaxone in a virtual human population using modeling and simulation. To the best of our knowledge, this is the first study that used the PBPK modeling and simulation to predict ceftriaxone exposure in the CKD patient population based on the degree of severity and physiological needs. The performance of the developed ceftriaxone PBPK model was verified visually, where the observed data contained within the constructed 5th to 95th predictive interval, and statistically according to values of the predicted-to-observed ratio, and all values fell within the predefined error range.

There has been widespread debate on the pharmacokinetics and appropriate dosing regimens of ceftriaxone in critically ill patients and those with various degrees of renal impairment. In a pharmacokinetic analysis of data obtained from critically ill patients infused with 2 g ceftriaxone once daily over 30 min, Joynt et al. (2001) concluded that ceftriaxone may be accumulated in patients with renal failure in comparison to those with intact renal function. It has been found that elimination half-life was 3-fold higher in patients with moderate or severe renal failure, and there was 50% reduction in clearance (Joynt et al., 2001). Moreover, another two pharmacokinetic studies demonstrated the accumulation of ceftriaxone in critically ill patients with moderate to severe renal failure (Heinemeyer et al., 1990; Van Dalen and Vree, 1990). The results from our PBPK modeling and simulation confirm what the abovementioned studies found in relation to the accumulation of ceftriaxone in patients with moderate and severe renal failure. In addition, we simulated the plasma concentration of 2 g intravenous ceftriaxone as being taken once daily (2 g every 24 h) or in a divided dosing scheme (1 g every 12 h). We found that the divided dosing scheme accumulated even more than single dosing regimen, illustrating the appropriateness of the single dose regimen. This finding is comparable to that found by TI et al. (1984). The investigators concluded that in patients with severe renal impairment, a once daily dosage regimen is feasible in compare to the 12-h dosage regimen (Ti et al., 1984). Furthermore, Stoeckel and Koup. (1984) found that a large single dose of ceftriaxone is favored rather than divided dose in case of renal insufficiency, despite no major accumulation was found in the patients (Stoeckel and Koup, 1984).

It has been argued that no dose adjustment was needed for ceftriaxone in case of renal failure due to the assumption that biliary clearance could make a balance on the total ceftriaxone clearance (Stoeckel and Koup, 1984). However, the contribution of biliary system into the overall clearance of ceftriaxone was not found to compensate the impairment of renal function (Heinemeyer et al., 1990; Grégoire et al., 2019). Moreover, the functional status of kidney was found to be one of the most important covariates that significantly impact the pharmacokinetic of ceftriaxone, and it has been recommended to be considered for the purpose of dosing adjustment (Bos et al., 2018; Grégoire et al., 2019).

In a recently published pharmacokinetic analysis of data obtained from three independently conducted studies, Heffernan et al. (2022) empirically described ceftriaxone pharmacokinetic parameters with taking into account both free and total concentration, and accordingly, optimized dosing regimens (Heffernan et al., 2022). The researchers mentioned that the dose of ceftriaxone should be adjusted based on renal function, albumin concentration, and minimum inhibitory concentration of the isolated pathogens. They recommended that ceftriaxone 1 g twice daily is generally suitable regimen for providing therapeutic exposure in patients with a normal renal function (creatinine clearance is around 100 mL/min), assuming that minimum inhibitory concentration is ≤0.25 mg/L. Importantly, because that patients’ overall clinical context (e.g., impaired renal function) should be taken into account, they mentioned that lower dose (1 g once daily) of ceftriaxone might be suitable for isolates with low minimum inhibitory concentration (≤0.125 mg/L). Furthermore, in critically ill patients with augmented renal clearance with or without hypoalbuminemia, the investigators recognized that there is a need for higher doses of ceftriaxone to achieve the therapeutic target exposure. In contrast to the empirical approach that they used, we described the ceftriaxone exposure using physiologically meaningful approach with more biologically relevant parameters and then we applied the model to predict exposure in patients with various degrees of CKD. We mainly focused on the applicability of the PBPK modeling in predicting ceftriaxone exposure in patients with renal impairment and subsequently determining the appropriate dose reduction that result in an exposure comparable to normal subjects.

The developed PBPK model precisely described an observed data obtained from a PK study conducted on both healthy and renal failure patients (Patel et al., 1984). Patel et al. (1984) examined the effects of renal failure on the pharmacokinetic of 1 g ceftriaxone infused over 15 min. They noted two-fold increase in half-life, and more than 50% decrease in plasma clearance, in comparison to young healthy population with intact renal function at the same dose. We tested our PBPK model for reproducing the results from this trial that was conducted on CKD patients with various degrees of severity. After stratifying the CKD patients by the exposure, we simulated the drug concentration after gradually reducing the dose that has been used in the clinical trial (1 g daily) to get a comparable exposure to healthy subjects. We found that 450–500 mg ceftriaxone in patients with moderate to severe renal failure resulted in a biological exposure that is comparable to the 1,000 mg that was given to healthy subjects in this trial. Thus, we concluded that 50% decrease in the dose for moderate to severe CKD is likely to provide the same exposure as seen in healthy individuals. In comparison to Heffernan et al. (2022) where the researchers depended on the minimum inhibitory concentration in the dosing recommendation, we provided our recommendation based on the comparability of total biological exposure in renally impaired patients to healthy individuals. It is important to note that while Heffernan et al. (2022) took into account both free and total concentration in their empirical model, our PBPK model is already accounting for this effect by incorporating the fraction of drug unbound as a drug-related parameter; Heffernan et al. (2022) generally recommended higher doses of ceftriaxone because the patients were critically ill, who are usually infected with more resistant bacteria and have lower albumin concentration (Heffernan et al., 2022). In contrast, Patel et al. (1984) recruited renally impaired patients otherwise free of clinical illness and they were not critically ill. The study was conducted to only characterize ceftriaxone kinetically in renally impaired patients, which is very important to understand the general trend in pharmacokinetic of ceftriaxone in renally impaired patients (Patel et al., 1984).

Patients with renal diseases are at a high risk of developing bacterial infections with increased resistance to many antibiotics (Berns and Tokars, 2002; Su et al., 2018; Wang et al., 2019). Simultaneously, there is a limited choice of antibiotics for treating these infections owing to their toxicity profiles. Ceftriaxone is commonly prescribed to patients with CKD at doses of 2 g/day or less, even though the median dose in patients diagnosed with ceftriaxone-induced toxicity was 1.7 g/day (Lacroix et al., 2021). A case report documented an association between a high therapeutic dose of ceftriaxone (2 g/12 h for 7 days) and the development of neurotoxicity in patients with End Stage Renal Disease (ESRD) (Hagiya et al., 2017). These adverse effects are present in the form of altered mental status, choreoathetosis, and myoclonus, and these adverse events disappear following drug withdrawal (Hagiya et al., 2017).

Other studies have demonstrated the influence of the kidney functional state on toxicity and how ceftriaxone led to side effects (Kim et al., 2012; Safadi et al., 2014; Tan and Tun, 2019; Yamada et al., 2020; Oyama et al., 2021). For instance, a case series reported that ceftriaxone treatment for a long period at high dosages was associated with the development of pseudolithiasis in patients with severe renal impairment who received dialysis (Oyama et al., 2021). In addition to pseudolithiasis, choreoathetosis was associated with ceftriaxone treatment (2 g/day) in ESRD patients (Tan and Tun, 2019). Moreover, a patient with CKD developed nonconvulsive status epilepticus after treatment with ceftriaxone (2 g/day) (Kim et al., 2012). Further, acute cholangitis and large pseudostones are produced in humans after kidney transplantation and are treated with ceftriaxone (Yamada et al., 2020). Other studies have found that ceftriaxone treatment at 2 g/day for 3 days causes encephalopathy in patients with ESRD (Safadi et al., 2014). Taken together, toxicity may develop in patients with ESRD treated with 2 g/day of ceftriaxone.

The variability in plasma concentration, protein binding, and other PK properties among individuals who received ceftriaxone (Patel and Kaplan, 1984; Popick et al., 1987; Schleibinger et al., 2015) play crucial roles in PBPK model system. A high percentage of ceftriaxone excreted renally in unchanged form, indicating that renal clearance is the rate-limiting step of ceftriaxone accumulation in patients with kidney diseases. Thus, PBPK can provide preliminary data regarding the expected ceftriaxone exposure in CKD patients. Extensive protein binding affects the kinetic behavior of ceftriaxone. This hypothesis was confirmed in a previous study demonstrating increased free ceftriaxone concentrations in the blood of patients with iatrogenic hypoalbuminemia (Mimoz et al., 2000). A different study found that ceftriaxone treatment at 1g/day resulted in values of 100% above the minimum inhibitory concentrations in patients with hypoalbuminemia and septic shock (Ulldemolins et al., 2021).

In summary, the kinetic properties of ceftriaxone after intravenous administration in healthy individuals and at various stages of CKD were successfully described using the current ceftriaxone PBPK model. Based on the generated AUC data, the model was used to suggest dosages for various CKD stages comparable to those in the healthy population. Clinicians should be aware about the increase in ceftriaxone exposure in patients with severe renal impairment, especially for diseases requiring high dosages of ceftriaxone. We outlined the current state-of-the-art of PBPK in drug investigations and provided guidance for future applications. In addition, we demonstrated that applying PBPK can help identify novel safety concerns and optimize dose regimens when conducting clinical trials with ceftriaxone in patients. Future PBPK studies are warranted to simulate further the antimicrobial compounds accumulations in the blood of patients with CKD and to recommend updated dosage regimens.

## Data Availability

The original contributions presented in the study are included in the article/Supplementary Material, further inquiries can be directed to the corresponding author.
