# Supplementary material for: Physiologically-based pharmacokinetic modeling for single and multiple dosing regimens of ceftriaxone in healthy and chronic kidney disease populations: a tool for model-informed precision dosing
Source: Front Pharmacol. 2023 Jul 20;14:1200828. doi: 10.3389/fphar.2023.1200828 (PMC10398570; doi:10.3389/fphar.2023.1200828)
Supplement: Supplementary file 1 [file DataSheet1.PDF]

A)

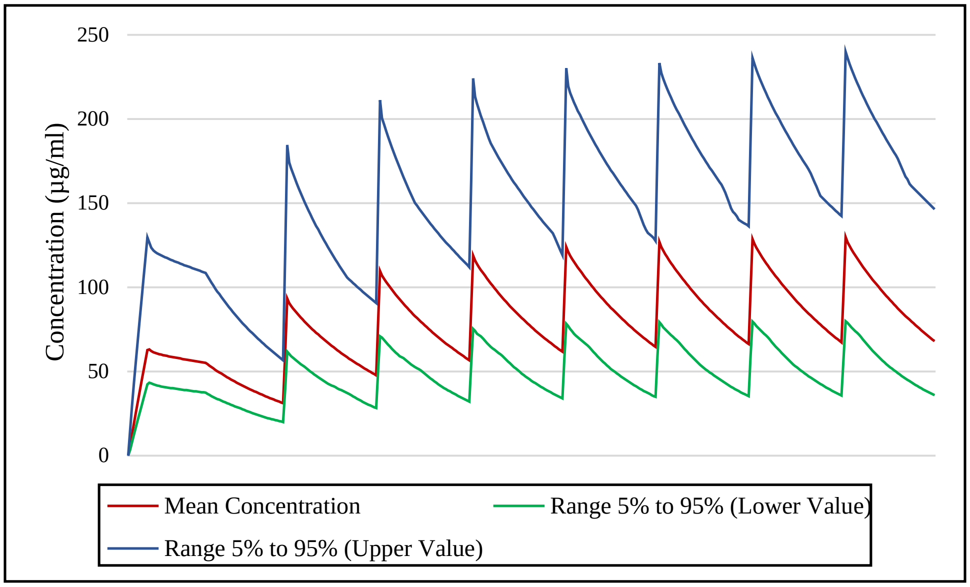

250
200
150
100
50
0
Concentration (µg/ml)
Mean Concentration
Range 5% to 95% (Lower Value)
Range 5% to 95% (Upper Value)

B)

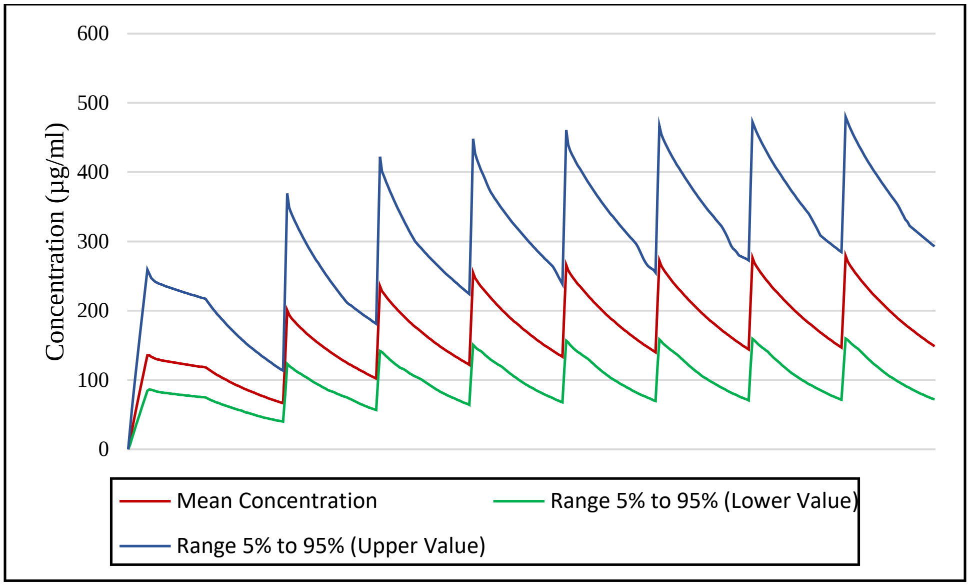

600
500
400
300
200
100
0
Concentration (µg/ml)
Mean Concentration
Range 5% to 95% (Lower Value)
Range 5% to 95% (Upper Value)

C)

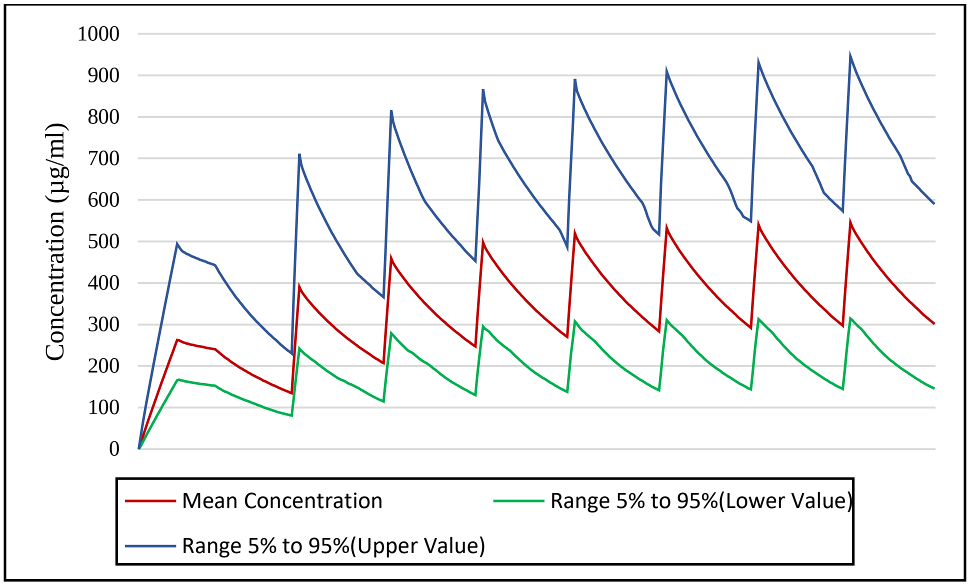

D)

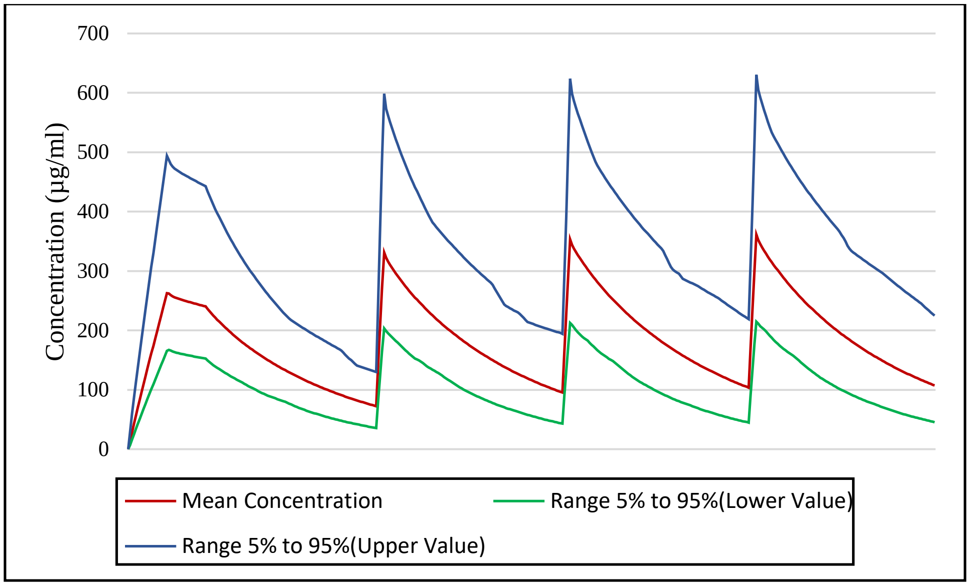

Figure 1S. A) Simulation of ceftriaxone 0.5g q12h in adult severe CKD population. B) Simulation of ceftriaxone 1g q12h in adult severe CKD population. C) Simulation of ceftriaxone 2g q12h in adult severe CKD population. D) Simulation of ceftriaxone 2g q24h in severe CKD population.
